# Global Leadership Initiative on Malnutrition-Diagnosed Malnutrition in Lung Transplant Candidates

**DOI:** 10.3390/nu16030376

**Published:** 2024-01-27

**Authors:** Alfonso Calañas-Continente, Jesús Gutiérrez-Botella, Julia García-Currás, Mª Jesús Cobos, José Manuel Vaquero, Aura Herrera, Mª José Molina, Mª Ángeles Gálvez

**Affiliations:** 1 Department of Endocrinology and Nutrition, University Hospital Reina Sofia, Avenida Menendez Pidal s/n, 14004 Cordoba, Spain; aurad.herrera.ssspa@juntadeandalucia.es (A.H.); cmmerinomjmolina@hotmail.com (M.J.M.); mariaa.galvez.sspa@juntadeandalucia.es (M.Á.G.); 2 Biostatech Advice Training and Innovation in Biostatistics, SL. Edificio Emprendia, Campus Vida s/n, 15782 Santiago de Compostela, Spain; jesus.gutierrez@biostatech.com (J.G.-B.); julia.garcia@biostatech.com (J.G.-C.); 3 Department of Pulmonary Medicine and Lung Transplantation, University Hospital Reina Sofia, Avenida Menendez Pidal s/n, 14004 Cordoba, Spain; mjesus.cobos.sspa@juntadeandalucia.es (M.J.C.); josem.vaquero.sspa@juntadeandalucia.es (J.M.V.)

**Keywords:** body composition, lung transplantation, malnutrition, nutrition assessment, reduced muscle mass, GLIM criteria

## Abstract

Background and aims: Malnutrition in lung transplantation (LT) candidates increases postoperative morbidity and mortality. Early diagnosis of malnutrition could attenuate adverse prognostic factors. This study aimed to assess the prevalence of nutritional risk and malnutrition using GLIM criteria in LT candidates and clinically characterize those with malnutrition. Methods: A prospective longitudinal study was conducted from 2000 to 2020 of LT candidates who underwent complete nutritional assessment (nutritional screening, anthropometry, bioelectrical impedance, blood laboratory tests and malnutrition diagnosis using GLIM criteria). Results: Obstructive diseases (45.6%), interstitial diseases (36.6%) and cystic fibrosis/non-cystic fibrosis bronchiectasis (15.4%) were the main conditions assessed for LT. Of the 1060 candidates evaluated, 10.6% were underweight according to BMI, 29% were at risk of malnutrition and 47% were diagnosed with malnutrition using GLIM criteria. Reduced muscle mass was the most frequent GLIM phenotypic criterion. Malnutrition was more prevalent in patients with cystic fibrosis/non-cystic fibrosis bronchiectasis (84.5%) and obstructive (45.4%) and interstitial (31.3%) diseases. GLIM criteria detected some degree of malnutrition in all diseases requiring LT and identified patients with higher CRP levels and worse respiratory function, anthropometric measurements and visceral protein and lipid profiles. Conclusions: LT candidates present a high prevalence of malnutrition using the GLIM algorithm. GLIM criteria detected malnutrition in all diseases requiring LT and defined patients with worse clinical-analytical profiles.

## 1. Introduction

In patients with advanced, severe lung disease, lung transplantation (LT) is a therapeutic option to improve their survival and quality of life. The most common conditions of these patients are chronic obstructive pulmonary disease (COPD), diffuse interstitial lung disease (ILD) and cystic fibrosis (CF) [1].

Advanced chronic lung diseases are associated with weight loss and protein–calorie imbalance, both of which are risk factors for malnutrition and increased morbidity. Lower fat-free mass in these patients is associated with worse clinical status and prognosis, regardless of body mass index (BMI) [2]. Severe or progressive malnutrition increases postoperative morbidity and mortality and is therefore considered a high-impact risk factor [3]. Early diagnosis of malnutrition allows the implementation of adequate nutritional treatment and minimizes the negative prognostic impact. Therefore, it is of interest to determine the prevalence of malnutrition in these patients in the most accurate manner possible [4,5,6,7,8,9].

Recently, the Global Leadership Initiative on Malnutrition (GLIM) updated and agreed on diagnostic criteria for malnutrition and published a consensus report representing experts from several international clinical nutrition societies [10]. The consensus report recommends a minimum set of clinically relevant diagnostic criteria that can be used in a variety of settings and patient populations. Since the publication of the report, GLIM criteria have been applied in numerous studies, but very few have examined the use of these criteria to determine the prevalence of malnutrition in LT candidates.

The objectives of this study were to (1) analyze the prevalence of nutritional risk and malnutrition in LT candidates using the GLIM framework for nutritional diagnosis; (2) establish the prevalence of malnutrition according to different lung diseases; and (3) clinically characterize the population of LT candidates with malnutrition.

## 2. Material and Methods

This was a prospective longitudinal cohort study of LT candidates who underwent nutritional assessment in our hospital from 2000 to 2020.

The Reina Sofia University Hospital of Cordoba, Spain, is one of seven nationally accredited LT centers in the country and has been operating since 1993. Each year, approximately 250 LT candidates are referred to our center for evaluation and more than 40 lung transplants are performed annually. The referral criteria for inclusion in the LT waiting list follow international guidelines that are updated periodically. Globally, LT should be considered in patients with a 50% higher risk of mortality at 2 years without transplantation and when the expected 5-year survival rate is greater than 80% considering normal graft function [3]. Due to the scarcity of donors and the high demand for LT, candidates must undergo an exhaustive multidisciplinary assessment to optimize their outcomes. These assessments aim to evaluate indications for transplantation and the presence of comorbidities or risk factors with prognostic impact, particularly those associated with nutrition.

The lung diseases subsidiary to transplantation in patients who underwent nutritional assessment were grouped into relatively homogeneous cohorts according to their functional behavior or pathophysiology. (1) Chronic obstructive lung diseases: emphysema, chronic bronchitis and other chronic obstructive diseases (Langerhans cells histiocytosis, bronchiolitis obliterans, graft-versus-host disease). (2) Interstitial lung diseases (ILDs): idiopathic pulmonary fibrosis, idiopathic nonspecific interstitial pneumonia, respiratory bronchiolitis-associated interstitial lung disease, desquamative interstitial pneumonia, cryptogenic organizing pneumonia, acute interstitial pneumonia, idiopathic lymphoid interstitial pneumonia, idiopathic pleuroparenchymal fibroelastosis, unclassifiable idiopathic interstitial pneumonias, hypersensitivity pneumonitis, autoimmune ILDs and other ILDs, including lymphangioleiomyomatosis, sarcoidosis, drug-associated ILDs, vasculitis/granulomatosis ILDs, proteinosis, alveolar microlithiasis, pneumoconiosis and other rare ILDs. (3) Cystic fibrosis (CF) and non-cystic fibrosis bronchiectasis (NCFB). (4) Vascular lung diseases such as pulmonary arterial hypertension and other vascular diseases.

The same nutritional assessment protocol was applied to all patients and included the Malnutrition Universal Screening Tool (MUST) [11] following the previously validated protocol, anthropometric measurements, bioelectrical impedance and clinical analyses.

Regarding anthropometric measurements, we considered: (a) weight, which was calculated using a weighing scale adjusted to 0.1 kg (SECA 665, Hamburg, Germany); (b) height, which was measured with a stadiometer (Holtain Ltd., Crymych, UK); (c) triceps skinfold thickness (TSF), which was measured with a caliper (Holtain Ltd., Crymych, UK); and (d) arm circumference (AC), which was measured using flexible non-elastic tape according to conventional protocols. All measurements were taken by the same investigator in the non-dominant arm and the mean of three skinfold and circumference measurements was calculated. Mid-arm muscle circumference (MAMC) was calculated based on TSF and AC. Patients with a BMI of ≥25 kg/m^2^ or ≥30 kg/m^2^ were diagnosed as being overweight or obese, respectively.

Fat mass (FM) and fat-free mass (FFM) were measured using a tetrapolar bioelectrical impedance device with an impedance analyzer applying an alternating current of 800 mA at a frequency of 50 Hz (BIA InBody 770, Cerritos, CA, USA). The fat-free mass index (FFMI) was calculated in relation to height as FFM (kg)/h^2^ (m^2^). To define reduced muscle mass, the FFMI cut-off points recommended by ESPEN were used [12].

The following clinical analyses were performed on the candidates: hemogram, visceral proteins (albumin, prealbumin, transferrin and total proteins), lipid profile and C-reactive protein (CRP).

### 2.1. Diagnosis of Malnutrition Using the GLIM Criteria

The GLIM tool differentiates data into two categories: phenotypic and etiologic markers [10]. Malnutrition diagnosis was based on the presence of at least one phenotypic criterion (weight loss, low BMI and/or reduced muscle mass) in combination with at least one etiologic criterion (reduced food intake or assimilation and/or disease burden/inflammation) as indicated by the GLIM consensus.

The phenotypic criterion for weight loss was defined as unintentional weight loss of >5% within the past 6 months. For patients of Caucasian, African or unknown ethnicity, the phenotypic criterion for low BMI was defined as a BMI < 20 kg/m^2^ in patients < 70 years old and BMI < 22 kg/m^2^ in patients ≥ 70 years old. BIA and anthropometric measurements were used to define reduced muscle mass. A low FFMI according to ESPEN cut-off points (<15 kg/m^2^ in females and <17 kg/m^2^ in males) [12], or a low MAMC (less than 21 cm, less than the lower tercile (p30) or less than p15 for the study population) were considered indicative of low muscle mass.

Malnutrition severity grading was based on the phenotypic criteria for BMI and weight loss.

As regards the etiologic criteria for reduced food intake or assimilation, the following data were recorded: changes in dietary intake (compared to usual intake); the duration of these changes in weeks; type of diet (fasting, low-calorie liquid diet, full liquid diet, insufficient solid diet); anorexia; and the presence and duration of one or multiple nutrition impact symptoms, including gastrointestinal symptoms such as dysphagia, nausea, vomiting, abdominal pain and diarrhea.

As recommended by the GLIM Working Group, CRP was used as a supportive measure for the etiologic criterion of disease burden/inflammation [10,13], for which a cut-off of >5 mg/L was considered. However, the LT candidates had advanced chronic respiratory diseases with several grades of inflammation and, therefore, inherently met the disease burden/inflammation criterion as established in the GLIM consensus report. Given that the etiologic criterion of inflammatory disease was assumed to be positive in all patients [2,14,15], the LT candidates participating in the study were diagnosed as having malnutrition if they met a phenotypic criterion.

According to the ESPEN criteria, a definitive diagnosis of malnutrition is established if BMI is < 18.5 kg/m^2^. Alternatively, malnutrition can be established if the following two criteria are met simultaneously: (1) weight loss > 10% indefinite of time or > 5% in the past 3–6 months and (2) BMI < 20 kg/m^2^ (in patients < 70 years) or BMI < 22 kg/m^2^ (in patients ≥ 70 years) or low FFMI [16].

### 2.2. Statistical Analysis

Qualitative variables were expressed as relative and absolute frequencies. The groups were compared using the chi-squared test when the sample size was sufficiently large. When the expected frequencies were lower than 5, Fisher’s exact test was used. Quantitative variables were described according to their mean, median, interquartile range and standard deviation. These variables were described using measures of central tendency and dispersion: mean ± standard deviation or median (interquartile range), as appropriate. Two group comparisons were performed using the Student’s *t*-test when the assumptions of normality and homoscedasticity were met. Welch’s *t*-test was used when there was heteroscedasticity and the Wilcoxon–Mann–Whitney test was used when the assumption of normality was violated. When more than two groups were compared, either the ANOVA or the Kruskal–Wallis test was used depending on whether the assumption of normality was met or not, respectively.

#### 2.2.1. Percentile Estimation

To define the quantiles for different measures of muscle mass, reference tables were used [13]. As the 15th and 85th percentiles were not defined, estimations were made according to two approaches: (a) using the properties of the normal distribution when the percentiles came from a Gaussian distribution or (b) fitting a generalized additive model (GAM) with the percentiles and obtaining point estimations under the 15th and 85th percentiles.

#### 2.2.2. Comparison of Nutritional Diagnostic and Screening Methods

To compare concordance between the two diagnostic methods, Cohen’s kappa coefficient was used. To compare a screening method against a diagnostic one as a gold standard, accuracy (correct classifications), balanced accuracy, specificity, sensitivity and predictive values were calculated.

A *p*-value of <0.05 was considered significant for all the analyses. All analyses were performed using R Software Version 4.1 [17].

## 3. Results

In the 2000–2020 study period, 1060 candidates met the criteria for complete nutritional assessment (69.5% males; 30.5% females). The mean age was 51.2 ± 12.9 years (males: 53.3 ± 11.6; females: 46.3 ± 14.3).

The main lung diseases for which transplant assessment was requested were obstructive diseases (45.6%), followed by ILDs (36.6%) and CF/NCFB (15.4%). Vascular diseases were infrequent (2.4%) in the transplant candidates referred to our center (Table 1).

The median BMI was 24.8 (7.5) kg/m^2^. According to this variable, 41.4% of the sample was of normal weight, 10.6% was underweight, 33.2% was overweight and 14.9% was obese (the majority, 13.1%, presented class 1 obesity). Morbid obesity was not detected in the study population.

### 3.1. Prevalence of Nutritional Risk and Malnutrition

When the MUST screening tool was used, 29% of the candidates were identified as being at nutritional risk (medium risk: 12.6%; high risk: 16.4%). This method showed good performance when compared to the GLIM diagnostic algorithm (sensitivity 0.69, specificity 0.86, PPV 0.65 and NPV 0.88). Globally, the MUST tool had a balanced accuracy of 0.78.

Of the total patients, 47% were diagnosed with malnutrition according to the GLIM criteria, most of whom presented moderate malnutrition (severe: 17.2%; moderate: 29.8%). When the criteria for reduced muscle mass were used, the prevalence of malnutrition was 35.8% according to FFMI (severe: 12.5%), 35.7% according to MAMC < 21 cm (severe: 13.8%), 33.1% according to MAMC < p15 (severe: 13.1%) and 37.5% according to MAMC < p30 (severe: 13.1%). Good concordance was found between the four reduced muscle mass criteria (kappa coefficient > 0.7).

Taking the ESPEN diagnosis as a reference, the GLIM phenotypic criteria used to define reduced muscle mass showed a similar performance. The diagnostic accuracy of the GLIM algorithm using the different definitions of reduced muscle mass versus the ESPEN diagnostic criteria is shown in Table 2. The GLIM definition that best matches the ESPEN diagnostic criteria according to Cohen’s kappa value is MAMC < p15, MAMC < 21 cm and low FFMI, although all of them showed good predictive performance when using the ESPEN as a reference.

**Figure 1:**
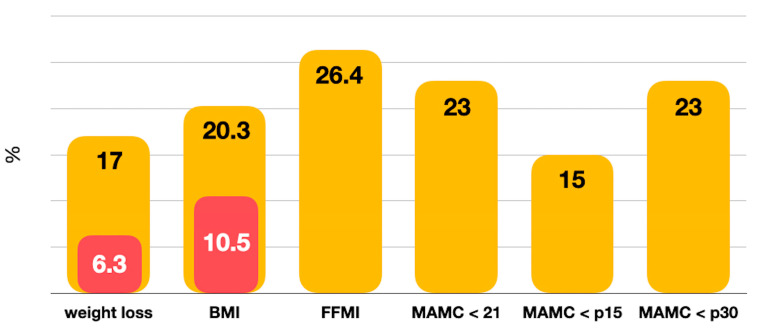
Distribution GLIM phenotypic criteria (in red % severity). BMI, body mass index; FFMI, fat-free mass index; MAMC, mid-arm muscle circumference.

The most frequent phenotypic criterion in this population was reduced muscle mass defined as low FFMI, as MAMC < 21 and as MAMC < p30, followed by low BMI and unintentional weight loss (Figure 1). Reduced food intake of any type was detected in 25.4% of the patients. Of the total candidates, 13.9% were receiving nutritional supplements at the time of pretransplant evaluation.

### 3.2. Malnutrition and Lung Disease

Patients with CF or NCFB showed a higher prevalence of malnutrition according to the GLIM framework (84.5%) (Table 3). According to the GLIM criteria, the prevalence of malnutrition was 45.4% for obstructive diseases and 31.3% for ILDs (*p* < 0.001). Malnutrition diagnosed by GLIM was detected in all the lung diseases included in the study.

**Figure 2:**
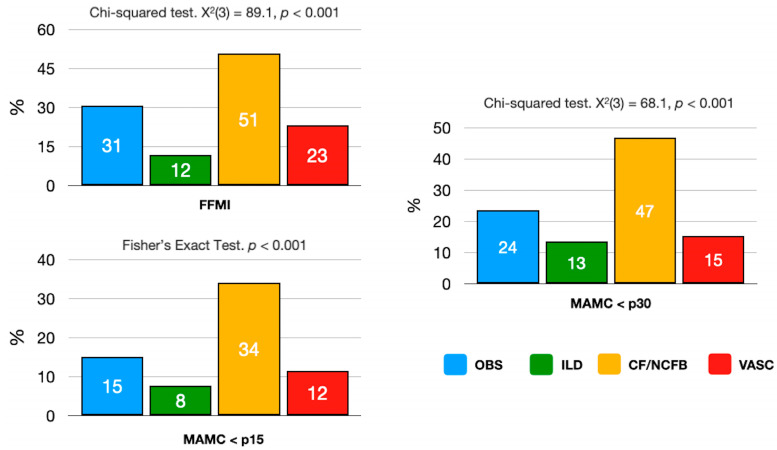
Prevalence of reduced muscle mass by disease. FFMI, fat-free mass index; MAMC, mid-arm muscle circumference. OBS, obstructive; ILD, interstitial lung disease; CF/NCFB, cystic fibrosis/non-cystic fibrosis bronchiectasis; VASC, vascular disease.

Reduced muscle mass was significantly more prevalent in LT candidates due to CF/NCFB, followed by candidates with obstructive and vascular diseases when using FFMI, MAMC < p30 or MAMC < p15 criteria (*p* < 0.001) (Figure 2).

### 3.3. Lung Transplant Candidates with Malnutrition

Malnourished LT candidates were significantly younger, predominantly female, presented worse pulmonary function and had lower weight, BMI, skinfolds, AC and MAMC, as well as a more unfavorable body composition (FM, FFM) than candidates with normal nutritional status. These patients also experienced significantly higher weight loss in the 6 months prior to assessment and lower levels of visceral proteins, total cholesterol, LDL cholesterol and triglycerides with higher CRP levels, although the increase in CRP was not significant (Table 4).

We analyzed the differences between malnourished lung transplant candidates diagnosed by GLIM criteria versus non-malnourished patients according to the group of pathologies subsidiary to transplantation. In general, there are no substantial differences in the profile of malnourished patients according to disease group, except for the variation in the statistical significance of variables related to respiratory function, visceral proteins and lipid profile. Tables S1–S4 provide additional information specific to each pathology (Tables S1–S4, Supplementary Materials).

## 4. Discussion

Our results show that the prevalence of malnutrition in the LT candidates is very high according to the GLIM criteria, affecting almost half of those included in the study (47%). Malnutrition is more prevalent in patients with CF/NCFB and in candidates with obstructive lung diseases (45.4%) and ILDs (31.3%). These consensus criteria for the diagnosis of malnutrition have allowed us to define a clinically vulnerable population with worse respiratory function, anthropometry and visceral protein and lipid profiles.

Nutritional assessment plays a crucial role in the multidimensional evaluation of LT candidates, although this is a very heterogenous population. Young patients with CF suffer pancreatic insufficiency, leading to malabsorption and extreme weight loss. In contrast, patients with obstructive diseases and ILDs are older and usually overweight or obese. This heterogeneity, as well as associated comorbidities, influences their nutritional needs and conditions their prognosis [18].

In transplantation candidates with advanced chronic lung diseases, significant weight loss is usually due to a calorie deficit and a state of chronic inflammation resulting in hypermetabolism. Loss of skeletal muscle mass and ventilatory inefficiency contribute to increased dyspnea, exertional intolerance and fatigue when eating, which causes further weight loss due to reduced caloric intake [19,20].

According to the GLIM algorithm, the first step in diagnosing malnutrition is to establish nutritional risk with a validated tool [15]. However, there is no standardized nutritional screening method for LT candidates. The screening method used in this study (MUST) is a good tool to exclude patients who do not present with malnutrition using the GLIM criteria. Negative nutritional screening with MUST enabled us to rule out malnutrition using the GLIM criteria in 88% of patients. MUST is very useful for excluding malnutrition, but less useful for diagnosing it.

Several studies have shown that MUST is a good predictor of hospital stays, mortality and discharge destination in different settings and has high concordance with other tools such as SGA [21,22]. The Academy of Nutrition and Dietetics concluded that MUST has high overall validity, moderate concordance and reliability and acceptable generalizability and quality of evidence [23]. In the GLIM algorithm, the use of screening as the first step is controversial [24,25,26,27], and various authors recommend performing a complete diagnosis including phenotypic and etiologic criteria in all patients with chronic lung disease, regardless of whether they have a positive screening result [24,25], as has been done in this study. In fact, our findings show that only 65% of truly malnourished patients diagnosed according to the GLIM criteria have a positive MUST screening result.

LT candidates have a high prevalence of malnutrition (47%), which is even higher when GLIM criteria are applied. However, if we had used the ESPEN criteria, the prevalence of malnutrition in the LT candidates would have been much lower (16%).

Published studies on the diagnosis of malnutrition using GLIM criteria in LT candidates are scarce. In a retrospective study of 112 patients, Emsley et al. [2] obtained a higher prevalence (59%). Although the populations are similar in both studies, the percentage of obesity in our study is almost 2.5 times higher (14.9 vs. 6%) and higher than the previously reported 12% in LT candidates [28]. Unlike the Australian study, our study included a higher percentage of patients with COPD (39.5 vs. 34%), who also presented the highest obesity rate among the LT candidates with obstructive diseases.

Other studies have reported a lower prevalence of malnutrition in patients with lung disease that ranges from 9–25% when definitions based on biochemical parameters are used or 11–43% for definitions based on low BMI [29,30,31,32,33]. As regards biochemical parameters, it should be noted that albumin and prealbumin levels are not useful as indirect measures of total body protein or total muscle mass. Considering these measures as surrogate markers of malnutrition is a gross simplification that should be avoided [34,35]. If we had used a BMI-based definition in this study, the proportion of underweight participants would have been 10.6%. This is much lower than the prevalence of malnutrition obtained with the GLIM or ESPEN criteria, and would have underestimated the actual prevalence. Moreover, the most frequent phenotypic criterion in this population is reduced muscle mass, not BMI.

The main guidelines and consensus documents for assessing LT candidates use BMI as a method to stratify and evaluate nutritional risk. Although low weight is an accepted risk factor, significant differences in survival are not always found in all candidates with a BMI of ≤17 kg/m^2^ [36,37]. The mechanisms underlying the adverse effects of high or low BMI on transplant outcomes remain unclear.

With the implementation of new methods to assess body composition, the use of BMI as the sole measure of nutritional status in LT candidates is questioned. BMI does not appear to be an accurate measure or surrogate of body composition [3], and the inconsistencies found in different studies may be due to the inability of this parameter to discriminate fat mass from muscle mass. Consequently, using BMI as the sole variable to determine nutritional status is questionable and inaccurate, especially in patients who need to gain or lose weight before being included on transplant waiting lists, a difficult task for patients with advanced lung disease. Therefore, the use of alternative anthropometric/body composition measures to complement BMI, such as those used in this study, could provide a more accurate and precise picture of the nutritional status of LT candidates.

The GLIM algorithm examined in this study to diagnose malnutrition is quite simple and can be used by a wide variety of health care professionals in their daily practice, even by those with limited training in nutritional assessment [38]. Furthermore, recent publications suggest that the GLIM diagnostic criteria are comparable to other established nutritional assessment tools for the diagnosis of malnutrition and its association with the risk of unfavorable events [39].

Similar to what has been reported for kidney transplant candidates, the most frequent phenotypic criterion in our population was reduced muscle mass, defined according to FFMI or MAMC [40]. Objective and validated body composition measures such as dual-energy X-ray absorptiometry, ultrasound, bioimpedance analysis, computed tomography or magnetic resonance imaging are currently recommended to diagnose low muscle mass [38]. In general, the use of these validated tools depends on their availability, the existence of standardized reference values and the practitioner’s own experience in measuring skeletal muscle mass or its related body compartments. When these premises are lacking, the use of anthropometry and physical examination as proxy measures has been proposed and agreed upon (92% agreement) [38,39]. In our study, simple anthropometric and bioimpedance analyses were employed to diagnose reduced muscle mass as a GLIM phenotypic criterion using different cut-off points. Regardless of which definition of low muscle mass was used, the prevalence of malnutrition was found to be similar (33.1–37.8%) and the four definitions of reduced muscle mass showed good agreement.

Currently, there is insufficient evidence to establish clear cut-off points for defining moderately or severely reduced muscle mass with the GLIM criteria [39]. For this reason, the severity of malnutrition was measured using the other two phenotypic criteria (weight loss and BMI), and found to be moderate in 29.8% and severe in 17.2% of our patients.

The GLIM nutritional algorithm does not establish in a universal and standardized way to assess the etiologic criterion of inflammation. Clinical (fever, leukocytosis, elevated basal energy expenditure) or biochemical (CRP, albumin and prealbumin) indicators can be used, which can help to assess the presence of inflammation [38]. Some studies have used the Glasgow prognostic score, which was originally proposed to assess risk in cancer patients undergoing surgery [41] and has been used to predict prognoses in other diseases such as idiopathic pulmonary fibrosis [42]. As in other studies on nutritional assessment in patients with chronic lung diseases [2,15], we assumed that all the patients assessed in our study met the etiologic criterion of moderate/severe chronic systemic inflammation given their advanced-stage lung disease.

Patients with CF or NCFB presented the highest prevalence of malnutrition, (84.5%) (Table 3). Malnutrition is extremely high in these patients due to numerous and very complex factors. Chronic bronchial infection increases protein caloric needs and favors anorexia. These patients may suffer dysgeusia, poor dietary habits acquired since childhood due to abnormal eating behaviors and various factors affecting digestion and food absorption such as exocrine pancreatic insufficiency, hepatopathy, bacterial overgrowth, intestinal inflammation, reduced bile acid production and intestinal dysmotility, among others [43,44]. In our survey, 30% of patients with CF or NCFB show one or multiple nutrition impact symptoms, such as anorexia, dysphagia, nausea, vomiting, abdominal pain and diarrhea, while in the rest of the pathologies evaluated, this symptomatology is less common and occurs in 15% of the patients. Anorexia is the most frequent symptom in all the groups of diseases evaluated.

The GLIM criteria enabled detecting malnutrition with a greater or lesser extent in frequency in all the subsidiary diseases requiring transplantation evaluated in this study. Likewise, the patients who met the GLIM criteria for malnutrition presented results of respiratory function, anthropometry, biochemistry and inflammation in accordance with their malnutrition status.

## 5. Limitations

More accurate and validated body composition assessment techniques, such as dual-energy X-ray absorptiometry or computed tomography, were not used in this study, which may have underestimated the prevalence of malnutrition. Additionally, strength and muscle function were not measured, which did not allow us to properly diagnose sarcopenia and sarcopenic obesity. These measurements were included in the nutritional assessment protocol of our center in 2020.

Although the GLIM criteria have provided a useful framework to give greater consistency to the diagnosis of malnutrition in these patients, the lack of consensus on the best way to measure muscle mass and classify the severity of muscle loss makes it difficult to evaluate this parameter in both research and the clinical setting. In our study, simple anthropometric measurements and bioimpedanciometry were used and obtained similar results, although they are probably lower than expected.

## 6. Strengths

One of the greatest strengths of our study is the large sample size, which ensures greater statistical power when analyzing the data. The fact that the same team of professionals performed the anthropometric and bioimpedance measurements over the study period may have prevented confounding effects in the analysis and permitted us to obtain more robust statistical conclusions.

## 7. Conclusions

The GLIM algorithm shows a high prevalence of malnutrition in LT candidates, affecting almost one out of every two patients evaluated. Malnutrition was more prevalent in patients with cystic fibrosis/non-cystic fibrosis bronchiectasis, obstructive and interstitial diseases. In our study, the GLIM criteria allowed us to detect malnutrition in all the diseases requiring LT and to define a population with higher CRP levels and worse respiratory function, anthropometric measurements and visceral protein and lipid profiles.

## 8. Clinical Implications

The use of the GLIM criteria in LT candidates may prevent underestimating the prevalence of malnutrition in these patients and allow targeted and early nutritional intervention that can have a potential impact on their prognosis. However, further studies are needed to validate the criteria in this and other patient populations.

The use of simple anthropometric measures to detect reduced muscle mass as a GLIM phenotypic criterion is adequate to diagnose malnutrition in this population. However, the use of BMI as the sole method to stratify and assess nutritional risk in LT candidates is highly questionable.

The inclusion of more precise morphofunctional nutritional variables in the nutritional evaluation protocol of our hospital based on these results, as well as a more regular and continuous follow-up of LT candidates at nutritional risk or with malnutrition, opens the possibility of establishing pre-transplantation variables predictive of post-transplantation outcomes.

## Figures and Tables

**Table 1:** Lung diseases subsidiary to transplantation.

	n (%)
OBSTRUCTIVE Emphysema Chronic bronchitis Combined pulmonary fibrosis and emphysema Others: Langerhans cells histiocytosis, bronchiolitis obliterans, graft-versus-host disease	483 (45.6%)
INTERSTITIAL Idiopathic pulmonary fibrosis Desquamative interstitial pneumonia Idiopathic nonspecific interstitial pneumonia Respiratory bronchiolitis-associated interstitial lung disease Cryptogenic organizing pneumonia Acute interstitial pneumonia Idiopathic lymphoid interstitial pneumonia Idiopathic pleuroparenchymal fibroelastosis Unclassifiable idiopathic interstitial pneumonias Hypersensitivity pneumonitis Autoimmune interstitial lung diseases Others: lymphangioleiomyomatosis, sarcoidosis, drug-associated ILDs, vasculitis/granulomatosis ILDs, proteinosis, alveolar microlithiasis, pneumoconiosis and other rare ILDs	388 (36.6%)
CYSTIC FIBROSIS NON-CYSTIC FIBROSIS BRONCHIECTASIS	163 (15.4%)
VASCULAR Pulmonary arterial hypertension Others	26 (2.4%)
	1060

ILDs, interstitial lung diseases.

**Table 2:** Diagnostic accuracy of different definitions of reduced muscle mass.

	Balanced Accuracy	Sensitivity	Specificity	PPV	NPV	Cohen’s Kappa
GLIM (FFMI) vs. ESPEN	0.882	1	0.763	0.417	1	0.483
GLIM (MAMC < 21 cm) vs. ESPEN	0.888	1	0.775	0.456	1	0.523
GLIM (MAMC < p15) vs. ESPEN	0.899	1	0.797	0.469	1	0.544
GLIM (MAMC < p30) vs. ESPEN	0.873	1	0.746	0.414	1	0.471

FFMI, fat-free mass index; MAMC: mid-arm muscle circumference; PPV: positive predictive value; NPV: negative predictive value.

**Table 3:** GLIM prevalence of malnutrition by disease group.

	GLIM
CF/NCFB (n = 155)	84.5% (n = 131)
OBSTRUCTIVE (n = 463)	45.4% (n = 210)
INTERSTITIAL (n = 377)	31.3% (n = 118)
VASCULAR (n = 24)	58.3% (n = 14)

CF, cystic fibrosis; NCFB, non-cystic fibrosis bronchiectasis.

**Table 4:** Characteristics of malnourished population by GLIM criteria.

	Malnourished	Not Malnourished	*p*
Age	46.7 (15.1)	55.1 (8.9)	<0.001
Male/Female	41.2%/60.1%	58.8%/39.9%	<0.001
FEV1 (%)	30.0 (17.1)	38.4 (17.9)	<0.001
TLC (%)	104.1 (42.1)	86.4 (39.9)	<0.01
pCO2 (mmHg)	46.3 (8.7)	43.5 (6.9)	<0.01
WL 6 months (yes)	84.2%	15.8%	<0.001
WL 6 months (kg)	5.1 (3.3)	2.5 (1.2)	<0.001
WL 6 months (%)	8.0 (4.6)	3.1 (1.1)	<0.001
Oral nutritional supplements (yes)	91%	8.6%	<0.001
Weight (kg)	58.2 (12.2)	78.0 (12.4)	<0.001
BMI (kg/m^2^)	21.2 (3.9)	27.9 (3.6)	<0.001
TSF (mm)	13.3 (6.8)	16.3 (6.3)	<0.001
SSF (mm)	12.9 (6.4)	20.1 (6.2)	<0.001
AC (cm)	25.4 (3.7)	30.9 (2.9)	<0.001
MAMC (cm)	21.2 (2.9)	25.8 (2.3)	<0.001
FM (kg)	13.9 (8.0)	24.4 (8.1)	<0.001
FFM (kg)	44.4 (8.3)	53.9 (8.7)	<0.001
Albumin (g/dL)	3.9 (0.5)	4.0 (0.4)	<0.01
Prealbumin (mg/dL)	22.2 (7.4)	24.0 (5.8)	<0.001
Cholesterol (mg/dL)	179.4 (51.0)	198.5 (40.2)	<0.001
HDL cholesterol (mg/dL)	49.6 (16.3)	47.9 (16.8)	<0.001
LDL cholesterol (mg/dL)	111.1 (40.3)	124.6 (35.5)	<0.001
Triglycerides (mg/dL)	106.0 (50.8)	127.6 (57.2)	<0.001
CRP (mg/L)	12.8 (22.6)	9.0 (16.3)	NS

FEV1, forced expiratory volume; TLC, total lung capacity; WL, unintentional weight loss; BMI, body mass index; TSF, triceps skinfold thickness; SSF, subscapular skinfold thickness; AC, arm circumference; MAMC, mid-arm muscle circumference; FM, fat mass; FFM, fat-free mass; CRP, C-reactive protein. NS, not significant. Plus–minus values are means ± SD.

## Data Availability

Data are contained within the article and Supplementary Materials.

## Supplementary Materials

The following supporting information can be downloaded at: https://www.mdpi.com/article/10.3390/nu16030376/s1. Table S1: Characteristics of malnourished population by GLIM criteria in obstructive diseases; Table S2: Characteristics of malnourished population by GLIM criteria in interstitial lung diseases; Table S3: Characteristics of malnourished population by GLIM criteria in cystic fibrosis and non-cystic fibrosis bronchiectasis; Table S4: Characteristics of malnourished population by GLIM criteria in vascular diseases.

## Abbreviations

AC, arm circumference; BMI, body mass index; CF, cystic fibrosis; COPD, chronic obstructive pulmonary disease; CRP, C-reactive protein; FEV1, forced expiratory volume; FFM, fat-free mass; FFMI, fat-free mass index; FM, fat mass; GLIM, Global Leadership Initiative on Malnutrition; ILD, interstitial lung disease; LT, lung transplantation; MAMC, mid-arm muscle circumference; MUST, Malnutrition Universal Screening Tool; NCFB, non-cystic fibrosis bronchiectasis; NPV, negative predictive value; PPV, positive predictive value; SSF, subscapular skinfold thickness; TLC, total lung capacity; TSF, triceps skinfold thickness; WL, unintentional weight loss.
