# Supplementary material for: Global Leadership Initiative on Malnutrition-Diagnosed Malnutrition in Lung Transplant Candidates
Source: Nutrients. 2024 Jan 27;16(3):376. doi: 10.3390/nu16030376 (PMC10857078; doi:10.3390/nu16030376)
Supplement: Supplementary file 1 [file nutrients-16-00376-s001.zip › nutrients-2826356-supplementary.pdf]

nutrients

MDPI

Table S1. Characteristics of malnourished population by GLIM criteria in obstructive diseases.

	Obstructive Diseases (N = 483)		
	**Malnourished**	**Not Malnourished**	***p***
**N**	223	260	
**Age**	55.6 (7.4)	56.7 (7.3)	<0.05
**Male/Female**	68.2% / 31.8%	82.3% / 17.7%	<0.001
**FEV1 (%)**	25.7 (12.6)	24.4 (10.0)	NS
**TLC (%)**	125.3 (39.7)	119.9 (31.8)	NS
**pCO2 (mmHg)**	47.5 (9.4)	47.0 (8.0)	NS
**WL 6 months (yes)**	43.5%	8.0%	<0.001
**WL 6 months (kg)**	4.7 (3.2)	2.1 (0.8)	<0.001
**WL 6 months (%)**	7.6 (4.5)	3.0 (1.0)	<0.001
**Oral nutritional supplements (yes)**	11.7%	2.0%	<0.001
**Weight (kg)**	58.3 (10.5)	75.9 (12.1)	<0.001
**BMI (kg/m²)**	21.0 (3.0)	27.0 (3.4)	<0.001
**TSF (mm)**	12.9 (6.4)	15.0 (6.0)	<0.001
**SSF (mm)**	12.8 (5.7)	18.7 (5.4)	<0.001
**AC (cm)**	25.7 (3.0)	30.3 (2.8)	<0.001
**MAMC (cm)**	21.6 (2.4)	25.6 (2.2)	<0.001
**FM (kg)**	13.6 (6.8)	22.6 (7.7)	<0.001
**FFM (kg)**	44.8 (7.6)	53.6 (8.2)	<0.001
**Albumin (g/dL)**	4.0 (0.4)	4.1 (0.4)	NS
**Prealbumin (mg/dL)**	23.4 (6.2)	23.8 (5.3)	NS
**Cholesterol (mg/dL)**	198.6 (43.5)	198.8 (38.1)	NS
**HDL-cholesterol (mg/dL)**	54.4 (16.4)	49.9 (18.2)	<0.001
**LDL-cholesterol (mg/dL)**	122.2 (38.6)	124.1 (33.2)	NS
**Triglycerides (mg/dL)**	105.1 (44.1)	122.7 (57.6)	<0.001
**CRP (mg/L)**	7.7 (18.7)	9.8 (19.3)	<0.01

FEV1, forced expiratory volume; TLC, total lung capacity; WL, unintentional weight loss; BMI, body mass index; TSF, triceps skinfold thickness; SSF, subscapular skinfold thickness; AC, arm circumference; MAMC, mid-arm muscle circumference; FM, fat mass; FFM, fat-free mass; CRP, C-reactive protein. Plus-minus values are means ± SD.

Table S2. Characteristics of malnourished population by GLIM criteria in interstitial lung diseases.

	Interstitial Lung Diseases (N = 388)		
	**Malnourished**	**Not Malnourished**	***p***
**N**	122	266	
**Age**	52.3 (11.5)	55.4 (8.5)	<0.05
**Male/Female**	66.4% / 33.6%	74.1% / 25.9%	NS
**FEV1 (%)**	41.8 (16.4)	49.5 (14.9)	NS
**TLC (%)**	48.8 (14.4)	59.8 (22.9)	NS
**pCO2 (mmHg)**	43.7 (7.5)	41.0 (5.1)	NS
**WL 6 months (yes)**	53.9%	8.4%	<0.001
**WL 6 months (kg)**	6.2 (3.7)	3.0 (1.4)	<0.001
**WL 6 months (%)**	8.5 (4.6)	3.4 (1.0)	<0.001
**Oral nutritional supplements (yes)**	11.0%	0.8%	<0.001
**Weight (kg)**	65.5 (13.5)	80.3 (12.2)	<0.001
**BMI (kg/m²)**	23.7 (4.1)	28.8 (3.5)	<0.001
**TSF (mm)**	15.8 (7.3)	17.6 (6.3)	<0.01
**SSF (mm)**	16.3 (7.2)	21.5 (6.5)	<0.001

**AC (cm)**	27.3 (3.7)	31.5 (2.9)	<0.001
**MAMC (cm)**	22.2 (3.1)	26.0 (2.4)	<0.001
**FM (kg)**	19.2 (9.2)	26.4 (8.0)	<0.001
**FFM (kg)**	46.1 (8.8)	54.2 (9.1)	<0.001
**Albumin (g/dL)**	3.9 (0.4)	4.0 (0.4)	NS
**Prealbumin (mg/dL)**	25.1 (8.6)	24.6 (6.0)	NS
**Cholesterol (mg/dL)**	198.8 (39.9)	201.1 (40.4)	NS
**HDL-cholesterol (mg/dL)**	49.0 (14.0)	46.4 (15.2)	<0.05
**LDL-cholesterol (mg/dL)**	125.6 (31.9)	126.7 (37.0)	NS
**Triglycerides (mg/dL)**	132.1 (63.6)	136.6 (56.9)	NS
**CRP (mg/L)**	12.2 (17.3)	8.3 (13.8)	NS

FEV1, forced expiratory volume; TLC, total lung capacity; WL, unintentional weight loss; BMI, body mass index; TSF, triceps skinfold thickness; SSF, subscapular skinfold thickness; AC, arm circumference; MAMC, mid-arm muscle circumference; FM, fat mass; FFM, fat-free mass; CRP, C-reactive protein. Plus-minus values are means ± SD.

Table S3. Characteristics of malnourished population by GLIM criteria in cystic fibrosis and non-cystic fibrosis bronchiectasis.

	**CF/NCFB (N = 163)**		
	**Malnourished**	**Not Malnourished**	***p***
**N**	137	26	
**Age**	28.4 (10.3)	40.50 (10.50)	<0.001
**Male/Female**	47.5% / 52.5%	65.4% / 34.6%	NS
**FEV1 (%)**	25.8 (10.1)	29.3 (7.2)	NS
**TLC (%)**	100.7 (19.7)	119.0 (7.8)	NS
**pCO2 (mmHg)**	47.1 (6.9)	43.7 (3.6)	NS
**WL 6 months (yes)**	48.8%	13.6%	<0.01
**WL 6 months (kg)**	4.6 (2.8)	1.5 (0.9)	<0.05
**WL 6 months (%)**	8.3 (4.9)	2.3 (1.5)	<0.05
**Oral nutritional supplements (yes)**	67.9%	20.0%	<0.001
**Weight (kg)**	51.1 (8.9)	73.7 (14.4)	<0.001
**BMI (kg/m^2)**	19.0 (2.7)	25.8 (3.6)	<0.001
**TSF (mm)**	11.6 (6.3)	16.1 (5.9)	<0.01
**SSF (mm)**	9.8 (4.5)	18.0 (6.5)	<0.001
**AC (cm)**	23.1 (3.2)	30.4 (3.0)	<0.001
**MAMC (cm)**	19.5 (2.5)	25.3 (2.6)	<0.001
**FM (kg)**	9.4 (5.0)	21.4 (7.9)	<0.001
**FFM (kg)**	41.9 (8.3)	54.3 (9.2)	<0.001
**Albumin (g/dL)**	3.8 (0.5)	3.9 (0.6)	NS
**Prealbumin (mg/dL)**	17.4 (5.9)	21.0 (7.5)	<0.05
**Cholesterol (mg/dL)**	135.2 (43.1)	165.2 (35.8)	<0.001
**HDL-cholesterol (mg/dL)**	42.8 (15.9)	46.3 (15.0)	NS
**LDL-cholesterol (mg/dL)**	78.7 (32.6)	101.7 (25.8)	<0.001
**Triglycerides (mg/dL)**	84.4 (36.9)	82.0 (27.5)	NS
**CRP (mg/L)**	23.5 (29.9)	10.5 (9.6)	<0.05

CF, cystic fibrosis; NCFB, non-cystic fibrosis bronchiectasis; FEV1, forced expiratory volume; TLC, total lung capacity; WL, unintentional weight loss; BMI, body mass index; TSF, triceps skinfold thickness; SSF, subscapular skinfold thickness; AC, arm circumference; MAMC, mid-arm muscle circumference; FM, fat mass; FFM, fat-free mass; CRP, C-reactive protein. Plus-minus values are means ± SD.

Table S4. Characteristics of malnourished population by GLIM criteria in vascular diseases.

	Vascular Diseases (N = 26)		
	**Malnourished**	**Not Malnourished**	***p***
**N**	16	10	
**Age**	37.9 (11.8)	43.5 (9.7)	NS
**Male/Female**	37.5% / 62.5%	50.0% / 50.0%	NS
**FEV1 (%)**	98.0 (17.0)	69.9 (-)	NS
**TLC (%)**	98.0 (18.4)	-	-
**pCO2 (mmHg)**	30.5 (5.0)	-	-
**WL 6 months (yes)**	66.7%	0.0%	<0.05
**WL 6 months (kg)**	3.8 (2.8)	-	-
**WL 6 months (%)**	5.2 (4.0)	-	-
**Oral nutritional supplements (yes)**	6.3%	0.0%	NS
**Weight (kg)**	64.0 (15.2)	79.7 (11.0)	<0.01
**BMI (kg/m²)**	23.5 (5.5)	29.3 (3.0)	<0.01
**TSF (mm)**	16.3 (7.8)	18.9 (5.1)	NS
**SSF (mm)**	14.4 (8.7)	23.4 (5.8)	<0.01
**AC (cm)**	26.9 (6.4)	32.0 (3.2)	<0.01
**MAMC (cm)**	21.8 (5.3)	26.1 (3.0)	<0.05
**FM (kg)**	16.1 (9.1)	24.9 (7.7)	<0.05
**FFM (kg)**	46.1 (10.3)	54.9 (11.0)	NS
**Albumin (g/dL)**	4.1 (0.4)	4.0 (0.4)	NS
**Prealbumin (mg/dL)**	23.2 (5.9)	24.8 (5.6)	NS
**Cholesterol (mg/dL)**	145.7 (30.9)	208.5 (62.7)	<0.05
**HDL-cholesterol (mg/dL)**	36.2 (9.2)	38.2 (12.3)	NS
**LDL-cholesterol (mg/dL)**	86.4 (29.3)	142.2 (53.4)	<0.01
**Triglycerides (mg/dL)**	112.2 (47.5)	137.7 (40.1)	NS
**CRP (mg/L)**	5.6 (11.0)	4.4 (2.8)	NS

FEV1, forced expiratory volume; TLC, total lung capacity; WL, unintentional weight loss; BMI, body mass index; TSF, triceps skinfold thickness; SSF, subscapular skinfold thickness; AC, arm circumference; MAMC, mid-arm muscle circumference; FM, fat mass; FFM, fat-free mass; CRP, C-reactive protein. Plus-minus values are means ± SD.
